# Silencing of Long Noncoding RNA Growth Arrest–Specific 5 Alleviates Neuronal Cell Apoptosis and Inflammatory Responses Through Sponging microRNA-93 to Repress PTEN Expression in Spinal Cord Injury

**DOI:** 10.3389/fncel.2021.646788

**Published:** 2021-05-14

**Authors:** Yuanwu Cao, Chang Jiang, Haodong Lin, Zixian Chen

**Affiliations:** ^1^Department of Orthopedics, Zhongshan Hospital, Fudan University, Shanghai, China; ^2^Department of Orthopedic Surgery, Shanghai General Hospital, Shanghai Jiaotong University School of Medicine, Shanghai, China

**Keywords:** spinal cord injury, lncRNA GAS5, miR-93, ceRNA, PTEN

## Abstract

A secondary injury induced by a spinal cord injury (SCI) remains the main cause of devastating neural dysfunction; therefore, it has been the subject of focused research for many years. Long noncoding RNA (lncRNA) has been found to participate in the SCI process, and this finding presents a high potential for diagnosis and treatment; however, the role of lncRNA in a secondary injury induced by SCI remains unclear. The aim of this study was to investigate the regulatory effect of lncRNA growth arrest–specific transcript 5 (GAS5) in secondary injury during SCI. The SCI mice model and hypoxic cellular model were established to research the roles of lncRNA GAS5 during SCI. Reverse transcription quantitative polymerase chain reaction (qRT-PCR) was conducted to determine the expression levels of microR-93 (miR-93) and lncRNA GAS5. Western blot analysis of the apoptosis regulator protein and terminal deoxynucleotidyl transferase dUTP nick end labeling assay was conducted to evaluate neuron cell apoptosis. Basso, Beattie, and Bresnahan (BBB) scores were calculated to assess neurological function. Flow cytometry was used to determine neuron cell apoptosis. The associations among GAS5, miR-93, and the phosphatase and tensin homolog (PTEN) were disclosed using RNA immunoprecipitation (RIP) assay, RNA pulldown assay, and dual-luciferase reporter assay. QRT-PCR demonstrated that GAS5 was significantly upregulated in both the SCI mice and hypoxic cellular models. GAS5 knockdown suppressed neuron cell apoptosis and inflammatory response in the SCI mice model. Further studies have indicated that GAS5 functions as a competing endogenous RNA (ceRNA) by sponging miR-93 in neuronal cells. In addition, PTEN was a target of miR-93, and GAS5 knockdown exhibited its anti-apoptotic and anti-inflammatory effects through the miR-93/PTEN axis. These findings suggest that the GAS5/miR-93/PTEN axis may be a promising therapeutic target for SCI.

## Introduction

Spinal cord injury (SCI) is a traumatic event that can cause permanent motor and sensory deficits. SCI affects approximately 250,000–500,000 people worldwide each year (Singh et al., 2014). The pathophysiology of SCI is best described as primary uncontrollable mechanical injury and secondary controllable degeneration. Because of the complex nature of a secondary injury following the primary injury, considerable research efforts have focused on understanding the pathophysiology of the secondary damage. Various cellular inflammatory responses and apoptosis following SCI mediate delayed tissue degeneration and contribute to secondary damage of the spinal cord (Liu et al., 2015). Thus, reducing secondary damage following SCI is key for preventing the propagation of additional injury.

Long noncoding RNAs (lncRNAs) were identified as nonprotein-coding transcripts >200 nt long that retain limited protein-coding capacity (Cech and Steitz, 2014). LncRNAs have emerged as new targets for regulating various cellular processes after SCI, such as inflammatory response, metabolism, and apoptosis (Zhong et al., 2016). For example, Zhou et al. (2018) have found that MALAT1 contributes to the inflammatory response of microglia in rats following SCI. Zhang H. et al. (2018) have shown that silencing lncRNA BDNF-AS reduces neuron cell apoptosis in an SCI cell model. Ren et al. (2019) have shown that lncRNA TCTN2 overexpression improves neurological function in an SCI rat model. Growth arrest–specific transcript 5 (GAS5), localized at chromosome1q25, is found to be upregulated in the hypoxic/ischemic-injured neonatal brain and hippocampal neurons, and GAS5 silencing reduces hypoxia/ischemia-induced brain injury and improves the recovery of neurological function (Zhao et al., 2018). Moreover, GAS5 was also found to be highly expressed in neurons subjected to oxygen–glucose deprivation/reoxygenation, and knockdown of GAS5 protects against ischemia/reperfusion-induced brain damage and improves overall neurological functions *in vivo* (Zhang et al., 2019). To the best of our knowledge, the biological function of GAS5 and its underlying mechanisms in SCI have not been determined.

Numerous studies have demonstrated that lncRNAs exert their roles by functioning as a competitive endogenous RNA (ceRNA) for microRNAs (miRNAs) to liberate the suppression of miRNAs on their target mRNAs (Wang et al., 2010; Cao et al., 2017). It has been noted that GAS5 can also serve as a ceRNA of specific miRNAs and can then affect various physiological and pathological processes. For example, Dai et al. (2019) have shown that GAS5 promotes neuron cell apoptosis after traumatic brain injury (TBI) by sponging miR-335. Chen et al. (2018) have revealed that GAS5 acts as a ceRNA for miR-137 to block activation of the Notch1 signaling pathway; thereby, decreasing neuron cell survival in the ischemic stroke mice model; however, it is not clear whether GAS5 acts as a ceRNA for miRNAs in the development of SCI.

In the present study, we investigated the expression and function of GAS5 in both an SCI mice model and an SCI cell model and clarified the mechanisms of action. We found that silencing GAS5 alleviated neuron cell inflammation and apoptosis in the SCI mice by regulating the miR-93/PTEN axis, which allowed us to better understand the pathologies of this disease.

## Materials and Methods

### Animals

Male C57BL/6 mice (8–10 weeks old, 25–30 g each) were purchased from the Shanghai SLAC Laboratory Animal Company Limited (Shanghai, China). All mice were housed under standard conditions (12-h light/dark cycle, 21 ± 2°C, ~55% humidity) with free access to food and water. Animal procedures were approved by the Ethics Committee for Experimental Animals of Zhongshan Hospital, Fudan University, China.

### Model Establishment

Mice were anesthetized with an intraperitoneal injection of 50 mg/kg pentobarbital sodium (Sigma-Aldrich, St. Louis, MO, USA), followed by a laminectomy, as previously described (Gruner, 1992). Briefly, an incision was made in the skin following the medial dorsal line and reaching the aponeurotic and muscular planes to expose the posterior vertebral arches from T8 to T12. Under dissection using a stereomicroscope, a 3-mm-long laminectomy, encompassing the caudal end of the T10 vertebra and the rostral end of the T11 vertebra, was conducted. The Infinite Horizons impactor (Infinite Horizons, LLC, Lexington, KY, USA) was used to produce a contusion SCI using a force of 60 kdyn/cm^2^. The Basso, Beattie and, Bresnahan (BBB) score was used to assess the locomotor behavior 1, 3, 7, 14, 21, and 28 days after injury. Subsequently, the injured mice were sacrificed to collect their spinal cord specimens (a 10-mm segment containing the injury epicenter) for cresyl violet staining, Western blotting, quantitative reverse transcription polymerase chain reaction (qRT-PCR), terminal deoxynucleotidyl transferase dUTP nick end labeling (TUNEL) assay, and immunohistochemistry (IHC) staining were conducted 7 and 14 days after SCI.

### Animal Treatment

The mice were randomly divided into the following two groups: SCI and sham (*n* = 10 each group/time) to evaluate the locomotor activity using the BBB score method 1, 3, 7, 14, 21, and 28 days after injury. The sham and SCI groups (7 and 14 days after SCI, respectively) were used to assess the spared tissue, apoptosis, and apoptosis-related protein expressions using cresyl violet staining, TUNEL assay, and Western blotting. The mice that underwent all surgical procedures without injury were used as the sham group.

To assess the functions of GAS5 during SCI, the mice were randomly divided into the following three groups (*n* = 10/group): sham, SCI + lentivirus short-hairpin RNA (LV-shRNA), and SCI + LV-scramble. In the SCI + LV-shRNA and LV-scramble groups, the mice were subjected to SCI and the lentivirus vectors LV-shRNA and LV-scramble [10^5^ plaque-forming units (PFU)] were injected into the intrathecal space of the mice using a glass micropipette, as previously described (Zhou et al., 2018). ShRNA specific for GAS5 was cloned into the LV-3 vector (GenePharma, Shanghai, China), and referred to as LV-shRNA. The LV-3 vector inserted with nontargeting shRNA was used as the negative control (LV- scramble).

To determine whether miR-93 is involved in the function of GAS5 on SCI *in vivo*, the mice were also randomly divided into the following three groups (*n* = 10/group): LV-shRNA, LV-shRNA + antagomiR-NC, and LV-shRNA + antagomiR-93. In the LV-shRNA + antagomiR-93/antagomiR-NC group, 10^5^ PFU LV-shRNA and 100 nM antagomiR-93/antagomiR-NC (RiboBio, Guangzhou, China) were injected into the intrathecal space of the SCI mice using a glass micropipette at 0, 1, and 2 days beginning 15 min after SCI.

### qRT-PCR

Total RNA from spinal cord tissues was isolated using TRIzol (Invitrogen, Carlsbad, CA, USA) according to the manufacturer’s instructions. Reverse transcription was generated using the PrimeScript RT Reagent Kit (TaKaRa Biotech, Dalian, China). QRT-PCR was conducted using the SYBR Premix Ex Taq (TaKaRa, Tokyo, Japan) on an ABI Prism7500 Sequence Detection System (Thermo-Fisher Scientific). Glyceraldehyde 3-phosphate dehydrogenase (GAPDH) was used as an internal control for phosphatase and tensin homolog (PTEN), and U6 was used as an internal control for lncRNA GAS5 and miRNAs. The primers used were as follows: lncRNA GAS5 forward: 5′-TGGTTCTGCTCCTGGTAACG-3′, reverse: 5′-AGGATAACAGGTCTGCCTGC-3′; PTEN 5′-TCCCAGACATGACAGCCATC-3′, reverse: 5′-TGCTTTGAATCCAAAAACCTTACT-3′; GAPDH forward: 5′-TCAACGACCCCTTCATTGACC-3′, reverse: 5′-CTTCCCGTTGATGACAAGCTTC-3′; miR-93 forward: 5′-AGTCTCTGGCTGACTACATCACAG-3′, reverse: 5′-CTACTCACAAAACAGGAGTGGAATC-3′; U6, forward: 5′-CTCGCTTCGGCAGCACA-3′, reverse: 5′-GTCATACTCCTGCTTGCTGAT-3′. Analyses of gene expression were conducted using the 2^−ΔΔ^Ct method (Livak and Schmittgen, 2001).

### BBB Score

The locomotor activity of the mice was evaluated using the BBB locomotion scale (Liu et al., 2017). The locomotor function was observed and analyzed individually by two independent and well-trained investigators according to the BBB scales.

## Tunel

The apoptosis of spinal cord tissues was detected using the TUNEL assay (*in situ* Cell Death Detection Kit, Roche, South San Francisco, CA, USA) 7 and 14 days after SCI. Briefly, spinal cord specimens obtained in the above experimental procedures were fixed in 4% paraformaldehyde (PFA) at 4°C in phosphate buffered saline (PBS; pH 7.4) for 20 min, embedded in paraffin, and sectioned to a thickness of 4 μm. The tissue sections were then added into a permeabilization solution and incubated with a TUNEL reaction mixture for 60 min at 37°C. After counterstaining with 4′6′-diamidino-2-phenylindole (DAPI), TUNEL-positive cells were counted from five randomly selected areas using an inverted fluorescence microscope (HB050; Zeiss, Hamburg, Germany; ×200 magnification).

### Immunohistochemical Staining

Immunohistochemistry (IHC) was used to determine the expression levels of cleaved caspase-3 and PTEN. The spinal cord tissue sections obtained in the above experimental procedure were blocked with 5% bovine serum albumin (Boster, Wuhan, China) for 60 min at room temperature. After washing with PBS three times, the tissue sections were incubated in anticleaved caspase-3 (cat. no.9664, Cell Signaling, MA, USA, 1:2,000) and anti-PTEN (cat. no. 9188, Cell Signaling, 1:1,000) at 4°C overnight. Secondary antibodies (cat. no. 7074; Cell Signaling Technology 1:200) were added at room temperature for 60 min and then counterstained with diaminobenzidine for 5–15 min at room temperature. Finally, images were taken using a microscope with a digital camera (VHX-5000, Keyence Corporation, Osaka, Japan) at 200× magnification.

### Lesion Identification Using Cresyl Violet

The spared tissue area was measured using cresyl violet staining. Briefly, the spinal cord specimens from the mice were perfused with 4% PFA and cuts of spinal cord sections were made using a cryostat. Samples using every 40th section of the lesion site were stained with 0.5% cresyl violet for 1 h at room temperature and evaluated using Image-Pro Plus 6.0 (Media Cybernetics, USA).

### Nissl Staining for the Detection of Neuronal Injury

Nissl staining was performed to detect neuronal injury. Sections of the spinal cord were washed with PBS at pH 7.4, and then dried at 55°C for 3 h and immersed in 0.9% crystal violet at 37°C (Sigma-Aldrich; Merck KGaA) for 2 h. Subsequently, the tissues were dehydrated with 70, 80, 90, and 100% ethanol for 5 min, and mounted with neutral balsam. Observations were performed using a fluorescent microscope (100 μm; Eclipse 80i; Nikon Corporation). Image-Pro Plus 6.0 (Media Cybernetics, Rockville, MD, USA) was used to conduct densitometric analysis and to determine the mean analysis area of Nissl^+^ cells. An average value was obtained by observing six random fields from each of the 10 slices per mice.

### Cell Culture and Hypoxia Treatment

HT22 cells were obtained from the Chinese Academy of Medical Science (Beijing, China) and maintained in Dulbecco’s modified Eagle’s medium (Gibco, Thermo Fisher Scientific) with 10% fetal bovine serum (FBS, Gibco; Thermo Fisher Scientific). The cells were grown under 37°C and 5% CO_2_.

Hypoxia treatment was conducted on the HT22 cells as previously described (He et al., 2018; Macks et al., 2018). Briefly, the cells were cultured overnight at 37°C in 5% CO_2_, 1% O_2_, and 94% N_2_ and then subjected to hypoxic treatment in a controlled airtight hypoxic chamber (Plas Labs, USA) containing 3% O_2_, 5% CO_2_, and 92% N_2_ at 37°C for 12 h or incubated under normoxic conditions as controls.

### Cell Transfection

Using Lipofectamine 2000 (Invitrogen) following the manufacturer’s instructions and recommendations, 2 μg GAS5 expression vector, pcDNA-GAS5 and pcDNA3.1 vectors, and miRNAs comprising 50 nM miR-93 mimics, 50 nM mimics NC, 50 nM miR-93 inhibitor, and 50 nM inhibitor NC (GenePharma Co., Ltd., Shanghai, China) were transfected into HT22 cells. After 24 h, the HT22 cells in 12-well plates were treated in a hypoxic incubator containing 3% O_2_, 5% CO_2_, and 92% N_2_ at 37°C for 12 h or incubated under normoxic conditions as controls.

To explore the biological function of GAS5 in neuronal cell lines, HT22 cells were infected with LV-shRNA at multiplicity of infection (MOI) = 1 followed by hypoxia treatment.

### Cell Apoptosis and Caspase-3 Activity

The Annexin V-FITC/PI apoptosis detection kit (Abcam, Cambridge, UK) was used to detect cell apoptosis. Briefly, the 100,000 cells/well were seeded in a 6-well-plate. The treated cells were washed twice with PBS and stained with annexin V and propidium iodide (PI). After incubating in the dark for 15 min at room temperature, apoptosis was measured using a FACScan flow cytometer (Beckman Coulter, Inc., Brea, CA, USA) and analyzed using FlowJo 8.7.1 software^1^. Caspase-3 activity was assessed using a caspase-3 activity assay kit (BestBio, Shanghai, China) according to the manufacturer’s instructions.

### Enzyme-Linked Immunosorbent Assay

The culture supernatant was collected from 12-well plates and concentrations of inflammatory cytokines, including tumor necrosis factor alpha (TNF-α; cat. no. 42120-1), interleukin (IL)-6 (cat. no. D6050), and IL-1β (cat. no. 42400-1), were measured by ELISA (ELISA) using protocols supplied by the manufacturer (R&D Systems, Abingdon, UK).

Spinal cord samples were harvested at 3 days post SCI as previously describled (Chen et al., 2019), and homogenized in PBS and then centrifuged at 5,000× *g* at 4°C for 10 min. The protein expressions of TNF-α, IL-6, IL-1β, and IL-10 in the supernatant were detected using ELISA and the protocols supplied by the manufacturer (R&D Systems).

### RNA Immunoprecipitation

Binding between lncRNA-GAS5 and miR-93 was estimated using the RNA-Binding Protein Immunoprecipitation Kit (Millipore, Boston, MA, USA). Briefly, HT22 cells were lysed in RNA immunoprecipitation (RIP) lysis buffer and centrifuged for 15 min at 14,000 rpm. Then, 2 μl argonaute2 (AGO2) antibody or negative control (normal mouse IgG, Abcam) coupled with 10 μl magnetic beads were added into the cell supernatant and incubated for 4 h at 4°C. The expression levels of lncRNA GAS5 and miR-93 were then measured using qRT-PCR.

### RNA Pulldown Assay

A pulldown assay was conducted using a Pierce Magnetic RNA-Protein Pull-Down Kit (Thermo Fisher Scientific; Wang et al., 2015). Cellular lysates were incubated with 50 pmol of biotinylated GAS5 and 50 μl streptavidin agarose magnetic beads (Life Technologies) at 4°C for 3 h, followed by boiling in SDS buffer for another 10 min. The Ago2 protein levels were detected using Western blotting, while miR-93 expression levels were measured using qRT-PCR.

### Western Blotting

Total protein was extracted from spinal cord tissues or HT22 cells using RIRA lysis buffer (Thermo Fisher Scientific), and the protein concentrations were determined using a BCA protein assay kit (Beyotime, Shanghai, China). Thirty micrograms of protein per lane were size-fractionated in 12% sodium dodecyl sulfate polyacrylamide gel electrophoresis and electrophoretically transferred to a polyvinylidene fluoride membrane (Millipore, Mississauga, Canada). The membranes were blocked with 5% skimmed milk for 2 h at room temperature and then incubated with anti-caspase-3 (cat. no. 14214, 1:2,000), anti-cleaved caspase-3 (cat. no. 9661, 1:1,000), anti-cleaved PARP (cat. no. 5625, 1:1,000), anti-Bax (cat. no. 14796, 1:500), anti-Bcl-2 (cat. no. 4223, 1:1,000), anti-PTEN (cat. no. 9188, 1:1,000), and anti-β-actin (cat. no. 4970, 1:5,000) overnight at 4°C. After washing with PBS and Tween three times, the membranes were incubated with horseradish peroxidase–conjugated secondary antibody (cat. no. 7074, 1:2,000) for 1 h at room temperature. All antibodies were purchased from Cell Signaling Technology, Inc. The proteins were visualized using enhanced chemiluminescence, and the band intensity was evaluated using Image-Pro Plus 6.0 (Media Cybernetics, Rockville, MD, USA).

### Luciferase Activity

The binding sites of GAS5 and miR-93 were predicted using a bioinformatics website^2^. Reporter vectors under the control of the wild-type and mutant human GAS5 3′-UTR were constructed using the pmirGLO plasmids (Promega, Madison, WI, USA). HT22 cells were transfected with 200 ng/ml of these plasmids using Lipofectamine 2000 (Invitrogen) in the presence of 50 nM miR-93 mimics, miR-93 inhibitor, or miR-NC. Forty-eight hours after transfection, firefly luciferase activity was measured and normalized using *Renilla* luciferase activity.

The binding sites of miR-93 and PTEN were predicted using bioinformatics software^3^. Reporter vectors under the control of the wild-type and mutant human PTEN 3′-UTR were also constructed using pmirGLO plasmids (Promega). HT22 cells were transfected with 200 ng/ml of these constructs using Lipofectamine 2000 (Invitrogen) in the presence of 50 nM miR-93 mimics, miR-93 inhibitor, or miR-NC. Forty-eight hours after transfection, the firefly luciferase activity was measured and normalized using *Renilla* luciferase activity.

### Statistical Analyses

All data are presented as the mean ± standard deviation. Pairwise comparisons were analyzed using Student’s *t*-test. Comparisons among groups where there were differences between groups were analyzed using one-way analysis of variance followed by Tukey’s *post-hoc* test. *P* < 0.05 was considered a statistically significant difference.

## Results

### lncRNA GAS5 Was Upregulated in SCI Mice

**Figure 1:**
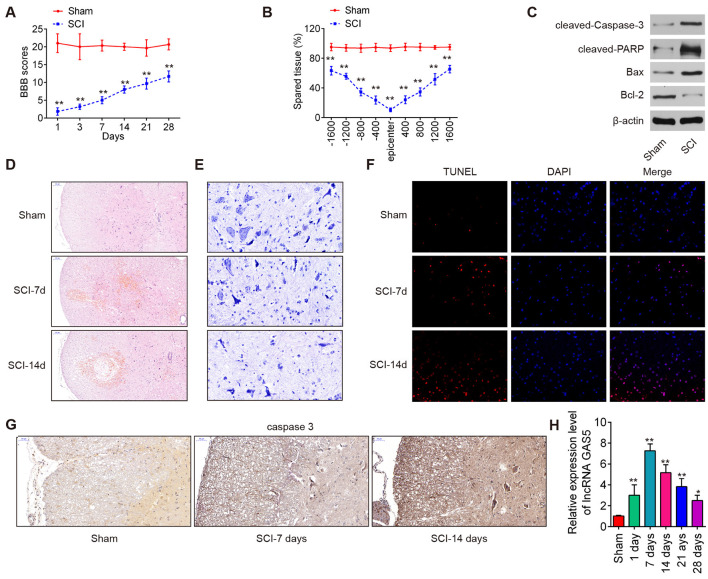
Long noncoding RNA (LncRNA) growth arrest–specific transcript 5 (GAS5) expression in spinal cord tissues of spinal cord injury (SCI) mice. **(A)** Basso, Beattie, and Bresnahan (BBB) scores for hindlimb locomotion in the SCI group and the sham group (*n* = 10 per group). **(B)** Lesion analysis at the rostral (−) and caudal (+) margins from the lesion epicenter as indicated using cresyl violet staining 14 days after the SCI model was established (*n* = 10 per group). **(C)** Protein expressions of Bcl-2, Bax, cleaved caspase-3, and cleaved PARP in spinal cord tissues measured using Western blot (*n* = 10 per group). **(D)** Representative pictures of H&E staining of the ventral horn of the mice spinal cord. The scale bar is 50 μm. **(E)** Representative images of Nissl staining of the spinal cord (magnification 400×). **(F)** Apoptosis in spinal cord tissues determined by terminal deoxynucleotidyl transferase dUTP nick end labeling (TUNEL) assay. **(G)** Immunohistochemistry (IHC) analysis of cleaved caspase-3 7 and 14 days after SCI. **(H)** Relative expression levels of long noncoding RNA (lncRNA) GAS5 in spinal cord tissues in the mice SCI model group and the sham group (*n* = 10 per group) using quantitative reverse transcription polymerase chain reaction (qRT-PCR). Notes: data represent the mean ± SD of three independent experiments. *p < 0.05, **p < 0.01 vs. sham group.

An SCI mice model was established in accordance with previous protocols, and the behavior of the mice was evaluated. As shown in Figure 1A, the SCI mice showed a slight spontaneous recovery of function during the experiment, accompanied by much lower BBB scores than that in the sham group. The tissues were stained with cresyl violet to assess the spared tissue following behavioral analyses. Compared with the sham group, there was less spared tissue in the injury epicenter of the SCI mice (Figure 1B). The levels of cleaved caspase-3, cleaved PARP, Bcl-2, and Bax in each group were examined using Western blot. The outcomes implied that compared with the sham group, the expressions of cleaved caspase-3, cleaved PARP, and Bax increased, while that of anti-apoptotic Bcl-2 decreased in the SCI group (Figure 1C). The pathological changes in the injured spinal cord were assessed by the HE staining. As shown in Figure 1D, the sham group displayed a well-defined border between gray matter and white matter, and neurons were abundant. In the injury group, tissue sections contained numerous red blood cells along the injury site with abundant inflammatory cells, particularly glial cell proliferation and satellitosis. Moreover, the injury group had a disordered spinal cord structure including indiscriminate structures between gray matter and white matter. Subsequently, surviving neurons in the lesioned spinal cord were identified by Nissl staining. A dramatic decrease in anterior horn neuronal loss was observed 7 and 14 weeks after the operation when we inspected Nissl bodies in the central area of the injured spinal cord (Figure 1E). In addition, apoptosis was evaluated using TUNEL staining 7 and 14 days after injury. There were more positively stained cells in the spinal cord tissues of the SCI mice than in the sham group (Figure 1F). IHC staining was applied to detect the level of cleaved caspase-3 in the spinal cord tissues. Compared with that in the sham group, there was a significant upregulation of caspase-3 in the spinal cord tissues of the SCI mice, especially upregulation in the gray matter of the spinal cord (Figure 1G). All data suggest that the SCI model was successfully constructed.

Finally, the expression levels of lncRNA GAS5 in the SCI tissues were determined using qRT-PCR at different time points. We observed that the expressions of lncRNA GAS5 in the SCI mice gradually increased and peaked at 7 days after injury and continued to significantly increase up to 28 days after injury (Figure 1H). These results indicated that lncRNA GAS5 may be involved in SCI pathogenesis.

### Knockdown of lncRNA GAS5 Improved Functional Recovery and Suppressed Neuron Cell Apoptosis and Inflammation in the SCI Mice Model

**Figure 2:**
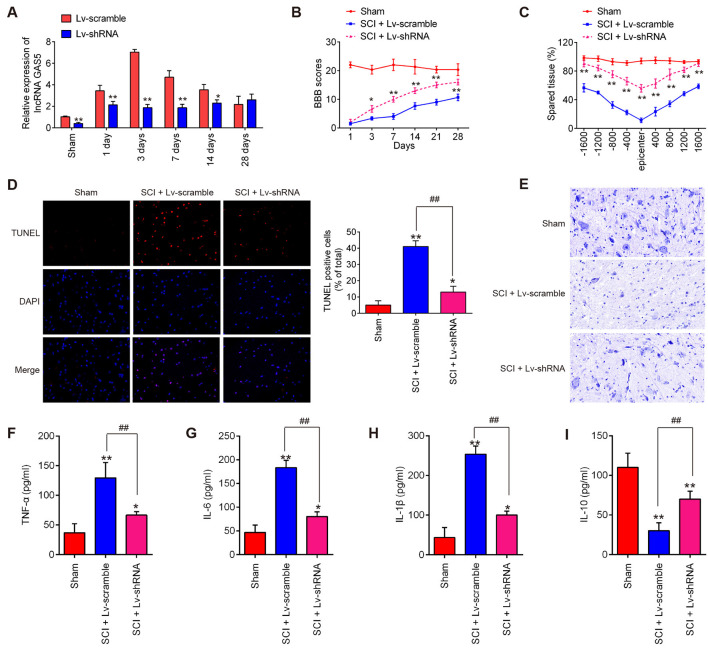
Knockdown of long noncoding RNA (lncRNA) GAS5 improves recovery of SCI mice by reducing apoptosis and inflammation. **(A)** Results of quantitative reverse transcription polymerase chain reaction (qRT-PCR) on expression levels of LncRNA GAS5 in spinal cord tissues 1, 3, 7, 14, and 28 days after lentivirus short-hairpin RNA (LV-shRNA) injection (*n* = 10 per group). **(B)** Basso, Beattie, and Bresnahan (BBB) score 1, 3, 7, 14, 21, and 28 days after SCI for all groups of mice (*n* = 10 per group). **(C)** Lesion analysis at the rostral (−) and caudal (+) margins from the lesion epicenter after cresyl violet staining 14 days after the SCI (*n* = 10/group/time). **(D)** Apoptosis determined using the terminal deoxynucleotidyl transferase dUTP nick end labeling (TUNEL) assay. **(E)** Representative images of Nissl staining of the spinal cord (magnification 400×). **(F–I)** Results of enzyme-linked immunosorbent assays (ELISA) on tumor necrosis factor (TNF)-α, interleukin (IL)-6, IL-1β, and IL-10 in spinal cord tissue grinding fluid from mice at 3 days post SCI (*n* = 10 per group). Data represent the mean ± SD of three independent experiments. *p < 0.05, **p < 0.01 vs. sham group; ^##^*p* < 0.01 vs. SCI + LV-scramble group.

To examine the functions of lncRNA GAS5 in SCI, we stably knocked down GAS5 by injecting LV-shRNA into the intrathecal space of the SCI mice. Analysis using qRT-PCR showed that *GAS5* levels notably decreased in the spinal cord tissues (Figure 2A). The locomotor activity in the mice in the shRNA group showed a remarkable recovery compared with that in the LV-scramble treatment group during the same period (Figure 2B). Results of the cresyl violet staining assay indicated that LV-shRNA could reduce lesion size in SCI mice, as observed by the increased amount of spared tissue compared with that in the LV-scramble-treated SCI mice (Figure 2C). Neuron cell apoptosis is also an important feature in SCI pathophysiology (Zhao W. et al., 2019). TUNEL staining was used to determine whether neuron cell apoptosis is associated with the protection of lncRNA GAS5 against SCI. As shown in Figure 2D; the positively stained cells in the SCI + LV-shRNA group had markedly decreased compared to those in the SCI group. Nissl staining results indicated a dramatic decrease in anterior horn neuronal loss in the central area of the injured spinal cord after SCI. However, LV-shRNA significantly promoted neuronal survival in the anterior horn compared to SCI group (Figure 2E), suggesting that GAS5 could promote neuron regeneration, which may be the exact reason for the improved functional recovery.

Inflammation is considered the most common phenomenon that immediately follows the secondary injury stage of SCI (Gao et al., 2018). It has been previously reported that GAS5 is involved in many types of injury models and could regulate subsequent neuron cell inflammation (Han et al., 2020; Li et al., 2020). Thus, we investigated the effects of lncRNA GAS5 on inflammation in SCI. The levels of pro-inflammatory cytokines, such as TNF-α, IL-6, and IL-1β, as well as immune-modulatory cytokine, IL-10 were detected using ELISA. As shown in Figures 2F–I, compared with the sham group, the levels of TNF-α, IL-6, and IL-1β significantly increased, but IL-10 was markedly decreased in the SCI group; whereas knockdown of lncRNA GAS5 reduced the expressions of these pro-inflammatory cytokines, while enhancing the levels of IL-10 induced by SCI. These results suggested that knockdown of lncRNA GAS5 promoted SCI recovery by suppressing apoptosis and inflammation.

### Knockdown of lncRNA GAS5 Suppressed Neuron Cell Apoptosis and Inflammation *In vitro*

**Figure 3:**
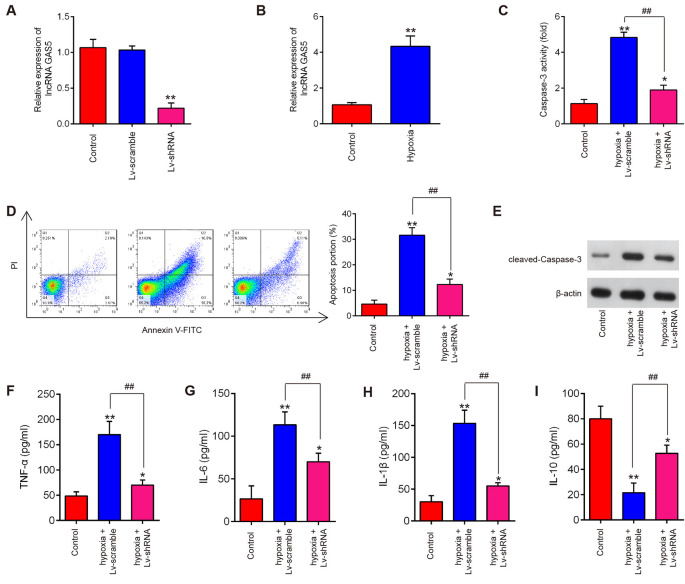
Silencing of long noncoding RNA (lncRNA) GAS5 reducing apoptosis and inflammation in HT22 cells under hypoxic conditions. **(A)** Transfected efficiency of lentivirus short-hairpin RNA (LV-shRNA) in HT22 cells as detected by quantitative reverse transcription polymerase chain reaction (qRT-PCR) analysis. **(B)** Expression levels of lncRNA GAS5 in HT22 cells under hypoxic conditions as measured by qRT-PCR. **(C)** The activity of caspase-3 measured using a caspase-3 activity assay kit. **(D)** Percentage of apoptotic neuronal cells detected using flow cytometry. **(E)** Protein expressions of cleaved caspase-3 as measured by Western blotting. **(F–I)** Levels of tumor necrosis factor (TNF)-α, interleukin (IL)-6, IL-1β, and IL-10 in cells as measured using enzyme-linked immunosorbent assay. Data represent the mean ± SD of three independent experiments. *p < 0.05, **p < 0.01 vs. control group; ^##^*P* < 0.01 vs. hypoxia + LV-scramble group.

SCI is always accompanied by tissue ischemia and hypoxia after the disruption of neural and vascular structures (Mautes et al., 2000; Sinescu et al., 2010). In fact, many experimental SCI models have indicated that long-lasting ischemia and hypoxia of the spinal cord results in microcirculation changes which are a key mechanism of secondary damage (Ellingson et al., 2019; Zhu et al., 2020). Following ischemia and hypoxia, a variety of inflammatory and cytotoxic mediators is released at the injured site, resulting in a fatal secondary neuronal degeneration in SCI. Many studies have used the hypoxia cell model to mimic spinal cord injury (Zhang H. et al., 2018; Zhang et al., 2021). In this study, HT22 cells were subjected to hypoxic treatment to construct a secondary SCI model *in vitro*. We then evaluated the roles of lncRNA GAS5 in neuron cell apoptosis and inflammation and manipulated the expression of GAS5 by infecting it with LV-shRNA. As shown by qRT-PCR, the expression of lncRNA GAS5 was successfully silenced (Figure 3A). In addition, we found that hypoxic treatment resulted in a significant increase in the expression levels of lncRNA GAS5 compared with that in the control group (Figure 3B). Subsequent experiments have revealed that silencing lncRNA GAS5 significantly reduced caspase-3 activity in hypoxia-treated HT22 cells (Figure 3C). Cell apoptosis was then further analyzed using this cell model. As expected, knockdown of lncRNA GAS5 significantly decreased the apoptotic rate in hypoxia-treated HT22 cells (Figure 3D). Meanwhile, the expression levels of cleaved caspase-3, an executioner caspase (Chakravarti et al., 2004), were assessed in this cell model. As shown in Figure 3E, those expression levels significantly increased following hypoxia treatment compared to that in the control, but significantly decreased by silencing lncRNA GAS5.

In addition, our results revealed that hypoxia treatment led to the notable increase of TNF-α, IL-6, IL-1β levels and remarkable reduction of IL-10 levels in HT22 cells, suggesting that hypoxia stimulation could trigger an inflammatory response in HT22 cells. However, silencing lncRNA GAS5 weakened the inflammatory response induced by hypoxia treatment (Figures 3F–I). Collectively, these results suggest that silencing lncRNA GAS5 improved the hypoxia-induced HT22 cell injury by suppressing neuron cell apoptosis and inflammation *in vitro*.

### lncRNA GAS5 Directly Targeted miR-93

**Figure 4:**
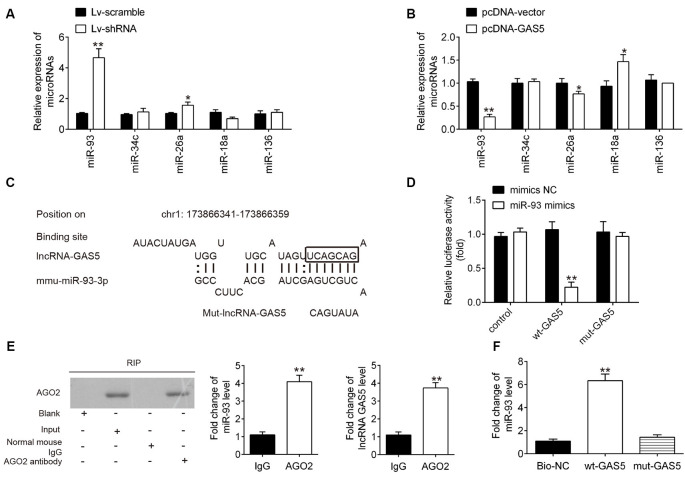
Interaction between long noncoding RNA (lncRNA) growth arrest–specific transcript 5 (GAS5) and miR-93. **(A,B)** Expressions of miR-93, miR-26a, miR-34c, miR-18a, and miR-136 as measured using quantitative reverse transcription polymerase chain reaction (qRT-PCR) analysis after lentivirus short-hairpin RNA (LV-shRNA) infection or pcDNA-GAS5 transfection in HT22 cells. Untreated cells served as control. Data represent the mean ± SD of three independent experiments. *p < 0.05, **p < 0.01 vs. LV-scramble group or pcDNA vector group. **(C)** Binding sites on lncRNA GAS5 and miR-93. **(D)** Relative luciferase activity of HT22 cells cotransfected with miR-93 mimics/NC and GAS5–3′UTR-WT or GAS5–3′UTR-MUT plasmid. **(E)** Ago2-RIP followed by Western blotting to assay miR-93 endogenously associated with GAS5. IgG was served as the control. Ago2 in proteins from Ago2-RIP assay was measured by Western blotting. The levels of GAS5 and miR-93 were measured by qRT-PCR. **p < 0.01 compared with the IgG group. **(F)** RNA pulldown assay was designed to explore the interaction between GAS5 and miR-93. Data represent the mean ± SD of three independent experiments. **p < 0.01 vs. negative control or beads group.

It has been reported that lncRNAs function as a ceRNA to regulate miRNA expression, thereby mediating SCI development (Gu et al., 2017). To further explore the mechanism by which GAS5 regulates neuron cell apoptosis and inflammation in an SCI model *in vivo* and *in vitro*, we conducted a bioinformatics analysis to identify GAS5-associated molecular regulators. By searching through StarBase v2.0, the online miRNA–lncRNA database, we observed that miR-93, miR-34c, miR-26a, miR-18a, and miR-136 have a very strong potential to bind GAS5. All of these miRNAs were reported to be associated with SCI (Jin et al., 2017; Wang et al., 2017; Zhang Y. et al., 2018; Xu et al., 2019). To verify this, we detected five miRNAs expression levels in the HT22 cells after GAS5 overexpression or knockdown. GAS5 overexpression or knockdown had the most significant influence on expression levels of miR-93; whereas, the other miRNAs were not affected (Figures 4A,B). Thus, we focused on miR-93 for further studies.

The binding sites between miR-93 and GAS5 are illustrated in Figure 4C. A luciferase assay was conducted to validate the relationship between miR-93 and GAS5. The results showed that miR-93 mimics reduced luciferase activity in the GAS5-WT group, but does not affect GAS5-Mut luciferase activity when compared with miR-93 NC (Figure 4D). The RIP assay showed that both miR-93 and GAS5 were enriched in the Anti-Ago2 group (Figure 4E). Figure 4F shows that miR-93 was pulled down by a biotin-labeled specific GAS5 probe, which indicates that miR-93 could directly bind to GAS5.

### PTEN Is a Direct Target of miR-93

**Figure 5:**
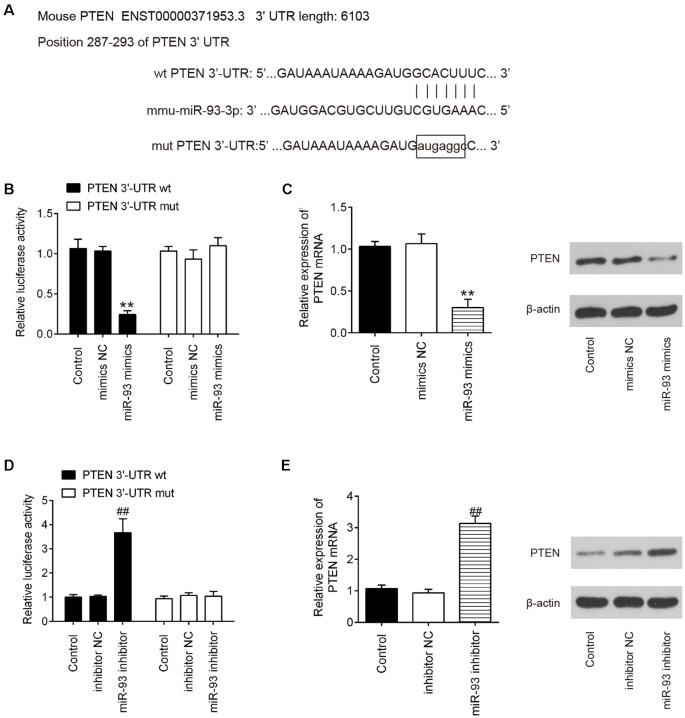
Phosphatase and tensin homolog (PTEN) is a direct target of miR-93 in HT22 cells. **(A)** Putative miR-93 binding sites on 3′-UTR of PTEN mRNA were predicted. **(B,D)** Relative luciferase activity of HT22 cells cotransfected with miR-93 mimics/inhibitor and PTEN-3′-UTR-WT or PTEN-3′-UTR-MUT plasmid. **(C,E)** mRNA and protein expression levels of PTEN in HT22 cells transfected with miR-93 mimics/inhibitor. Data represent the mean ± SD of three independent experiments. **p < 0.01 vs. mimics negative control (NC) group; ^##^*p* < 0.01 vs. inhibitor NC group.

Having confirmed that GAS5 could negatively regulate miR-93 expression, we then aimed to identify the main target genes of miR-93. TargetScan online tools were used to screen downstream target genes for miR-93; PTEN was identified as that target. The binding sites between miR-93 and PTEN are illustrated in Figure 5A. Next, dual-luciferase reporter assays were used to determine whether PTEN is directly targeted by miR-93 in HT22 cells. We observed that miR-93 overexpression obviously reduced the luciferase activity of pGL-PTEN-3′-UTR-WT; whereas, miR-93 knockdown increased that activity. There was no significant effect on luciferase activity when cotransfecting with PTEN containing mutated binding sites (Figures 5B,D). In addition, we observed using qRT-PCR and Western blotting that miR-93 overexpression dramatically decreased PTEN expression, while miR-93 knockdown significantly increased PTEN expression on mRNA and protein levels in HT22 cells (Figures 5C,E). These data suggest that PTEN may be a functional target of miR-93 in HT22 cells.

### lncRNA GAS5 Positively Regulated PTEN by Targeting miR-93 *In vitro* and *In vivo*

**Figure 6:**
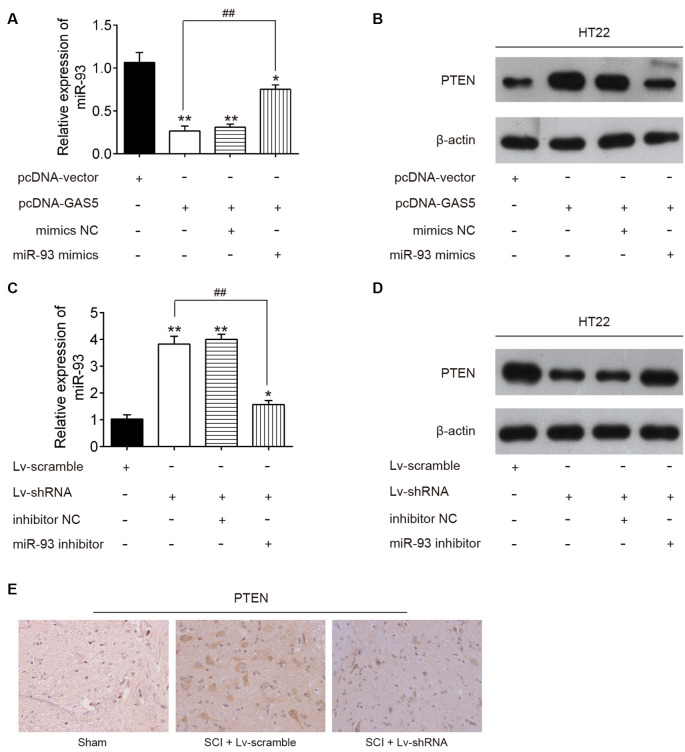
Long noncoding RNA (lncRNA) growth arrest–specific transcript 5 (GAS5) regulates the expression of phosphatase and tensin homolog (PTEN) through sponging miR-93. The pcDNA vector and pcDNA-GAS5 alone or together with miR-93 mimics or mimics negative control (NC) was transfected into HT22 cells. Twenty-four hours after transfection, the expression levels of miR-93 and PTEN protein levels in HT22 cells were measured by quantitative reverse transcription polymerase chain reaction (qRT-PCR) and Western blot analysis. **(A,B)** Data represent the mean ± SD of three independent experiments. *p < 0.05, **p < 0.01 vs. pcDNA-vector group; ^##^*p* < 0.01 vs. pcDNA-GAS5 group. **(C,D)** Lentivirus (LV)-scramble and lv-short-hairpin RNA (shRNA) alone or together with miR-93 inhibitor or inhibitor NC was transfected into HT22 cells; 24 h after transfection, the expression levels of miR-93 and PTEN protein levels in HT22 cells were measured using qRT-PCR and Western blot analysis. **(E)** The expression of PTEN was detected in spinal cord tissues of SCI mice after LV-shRNA injection.

To determine whether lncRNA GAS5 regulates PTEN by targeting miR-93 in neuron cells, HT22 cells were cotransfected with pcDNA-GAS5, pcDNA-GAS5 plus miR-93 mimics, LV-shRNA, or LV-shRNA plus miR-93 inhibitor. Notably, GAS5 overexpression reduced the expression levels of miR-93 and increased those of PTEN protein; these effects were reversed by overexpression of miR-93 (Figures 6A,B). Similarly, GAS5 silence increased the expression levels of miR-93 and reduced those of PTEN protein; however, miR-93 inhibitor reversed these effects (Figures 6C,D). Meanwhile, we measured the expression levels of PTEN in the spinal cord tissues of SCI mice using IHC staining. As expected, the expression levels of PTEN significantly increased in the SCI group, while the LV-shRNA injection obviously reduced those levels (Figure 6E). Collectively, lncRNA GAS5 could function as an miR-93 decoy to liberate the expression of PTEN.

### lncRNA GAS5 Inhibited Neuron Cell Apoptosis and Inflammation by Targeting miR-93

**Figure 7:**
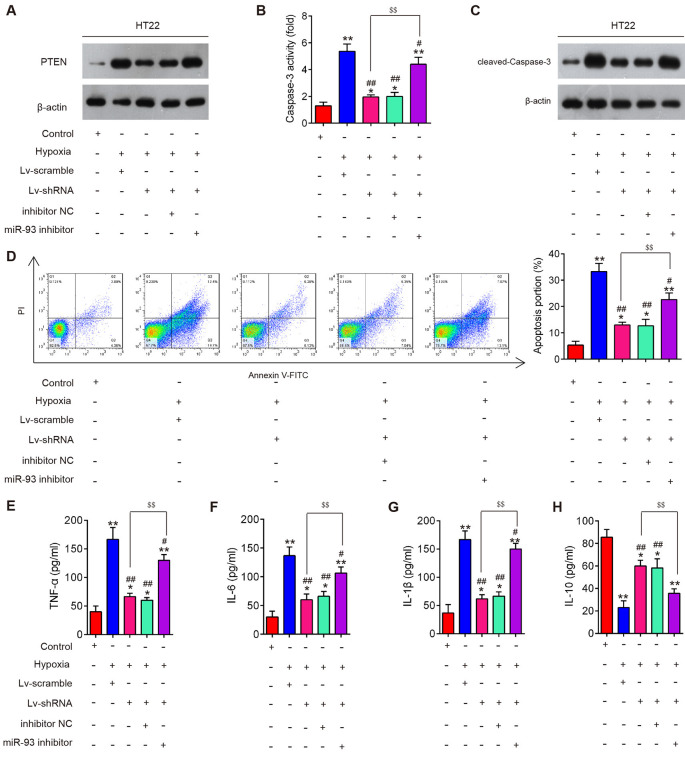
Silencing of long noncoding RNA (lncRNA) growth arrest–specific transcript 5 (GAS5) reduced apoptosis and inflammation by regulating miR-93 *in vitro*. HT22 cells were cotransfected with lentivirus short-hairpin RNA (LV-shRNA) and miR-93 inhibitor in HT22 cells, followed by hypoxia treatment. **(A)** The expression of phosphatase and tensin homolog (PTEN) in HT22 cell extracts in different groups was measured using Western blotting. **(B)** The activity of caspase-3 was measured using a caspase-3 activity assay kit. **(C)** Protein expression of cleaved caspase-3 was measured using Western blotting. **(D)** The percentage of apoptotic neuronal cells was detected using flow cytometry. **(E–H)** Levels of tumor necrosis factor (TNF)-α, interleukin (IL)-6, IL-1β, and IL-10 in neuronal cells were measured using enzyme-linked immunosorbent assay. Data represent the mean ± SD of three independent experiments. *p < 0.05, **p < 0.01 vs. control group; ^#^*p* < 0.05, ^##^*p* < 0.01 vs. hypoxia + LV-scramble group; ^$$^*p* < 0.01 vs. hypoxia + LV-shRNA group.

To explore whether miR-93/PTEN axis is involved in the inhibitory effects of silencing lncRNA GAS5 on hypoxia-induced neuron cell apoptosis and inflammation, HT22 cells were cotransfected with LV-shRNA and miR-93 inhibitor, followed by hypoxia treatment. As shown in Figure 7A, PTEN protein expression significantly increased in the HT22 cells after hypoxia treatment, but GAS5 knockdown markedly decreased this expression; however, this inhibitory effect was reversed by inhibiting miR-93. Meanwhile, we found that GAS5 knockdown reduced the activity of caspase-3 (Figure 7B) and the expression of cleaved caspase-3 (Figure 7C), as well as inhibited neuron cell apoptosis (Figure 7D) in HT22 cells under hypoxia treatment; whereas, these inhibitory effects were significantly reversed by inhibiting miR-93. In addition, we observed that GAS5 knockdown reduced the secretion of pro-inflammatory cytokines, such as TNF-α (Figure 7E), IL-6 (Figure 7F), and IL-1β (Figure 7G), but enhanced the levels of IL-10 (Figure 7H) in HT22 cells under hypoxia treatment; however, the inflammatory response induced by hypoxia treatment was also attenuated by inhibiting miR-93. All of these results suggest that lncRNA GAS5 inhibited cell apoptosis and inflammation by targeting miR-93.

### lncRNA GAS5 Knockdown Relieved Neuron Cell Apoptosis and Inflammation Through the miR-93/PTEN Axis in the Mice SCI Model

**Figure 8:**
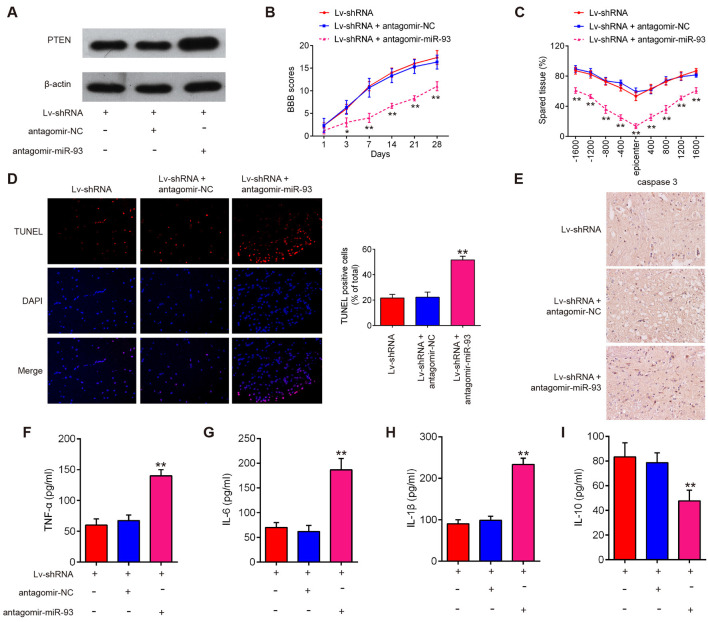
Knockdown of long noncoding RNA (lncRNA) GAS5 improves recovery of spinal cord injury (SCI) mice by regulating the miR-93/phosphatase and tensin homolog (PTEN) axis. The lentivirus vectors lentivirus short-hairpin RNA [LV-shRNA; 105 plaque-forming units (PFU)] and 100 nM antagomiR-93 were injected into the intrathecal space of mice SCI models using a glass micropipette. At the indicated time, the animals were sacrificed and the spinal cord was harvested for further experiments. **(A)** Expression of PTEN extracts in different groups as measured by Western blotting at 14 days post-SCI. **(B)** Basso, Beattie, and Bresnahan (BBB) scoring on hindlimb locomotor activity of SCI mice (*n* = 10 per group). **(C)** Lesion analysis at the rostral (−) and caudal (+) margins from the lesion epicenter using cresyl violet staining 14 days after SCI (n = 10 per group). **(D)** Apoptosis determined by terminal deoxynucleotidyl transferase dUTP nick end labeling (TUNEL) assay at 14 days post-SCI. **(E)** Immunohistochemistry (IHC) analysis of cleaved caspase-3 in spinal cord tissues at 14 days post-SCI. **(F–I)** Levels of tumor necrosis factor (TNF)-α, interleukin (IL)-6, IL-1β, and IL-10 in tissue grinding fluid from mice at 3 days post-SCI as measured using enzyme-linked immunosorbent assay (*n* = 10 per group). Data represent the mean ± SD of three independent experiments. *p < 0.05, **p < 0.01 vs. LV-shRNA group.

To determine whether GAS5 knockdown exhibited its anti-apoptotic and anti-inflammatory effects through the miR-93/PTEN axis, LV-shRNA and antagomiR-93 were injected into the intrathecal space of the mice in the SCI group. As shown in Figure 8A, PTEN protein expression markedly increased in the LV-shRNA plus antagomiR-93 group, compared with that in the LV-shRNA or LV-shRNA plus antagomiR-NC group. The BBB scores showed that knockdown of lncRNA GAS5 improved the functional recovery of the SCI mice, while this improvement was significantly attenuated by antagomiR-93 (Figure 8B). Using cresyl violet staining, we observed that the amount of spared tissue significantly decreased in the antagomiR-93 + LV-shRNA group compared with that in the LV-shRNA group (Figure 8C). The apoptosis and inflammatory responses were then reassessed. As shown in Figures 8D,E, we observed that apoptosis and the expression levels of caspase-3 that were suppressed in the SCI mice by lncRNA GAS5 knockdown were reversed by miR-93 inhibition. Similarly, the inhibitory effects of lncRNA GAS5 knockdown on the inflammatory response were abolished by miR-93 inhibition (Figures 8F–I). All of these data strongly confirm that the effects of lncRNA GAS5 on apoptosis and inflammatory responses are achieved through the miR-93/PTEN axis.

**Figure 9:**
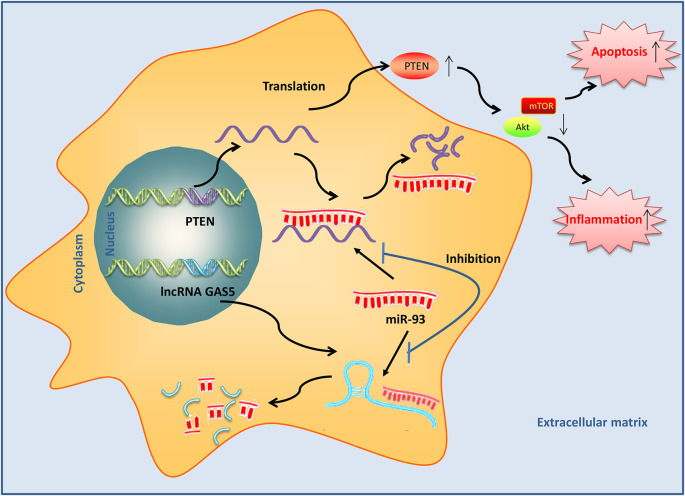
Long noncoding RNA (lncRNA) growth arrest–specific transcript 5 (GAS5) induced by SCI acts as an endogenous sponge of miR-93 to promote phosphatase and tensin homolog (PTEN) protein expression and subsequently inactivates PI3K/AKT, thus resulting in the induction of apoptosis and inflammatory response in SCI mice.

These results suggested that GAS5 upregulation could activate PTEN by binding with miR-93, inactivate the PI3K/AKT pathway, and subsequently lead to inducing the apoptosis and inflammatory response during SCI (Figure 9).

## Discussion

In the present study, GAS5 was upregulated in the spinal cord tissues of SCI mice. GAS5 silencing improved functional recovery and reduced the lesion size in the spinal cord in the SCI mice. Moreover, GAS5 knockdown inhibited SCI-induced cell apoptosis and inflammatory response *in vivo* and *in vitro* by regulating the miR-93/PTEN axis in the secondary injury after SCI. Our results suggest that GAS5 may be a new therapeutic target for SCI patients.

Several studies have revealed that GAS5 acts as a new regulator of apoptosis in animal and cell models after various types of injury. For example, Zhao J.-H. et al. (2019) have found that GAS5 knockdown suppresses neuron cell apoptosis in cerebral infarction (CI) mice by activating the Notch1 signaling pathway. Dai et al. (2019) have shown that GAS5 inhibition suppresses neuron cell apoptosis following TBI through the miR-335/rasa1 axis. Deng et al. (2020) have reported that GAS5 silencing suppresses neuron cell apoptosis and improves nerve injury after ischemic stroke by inhibiting DNMT3B-dependent MAP4K4 methylation. Notably, Wu et al. (2019) have demonstrated that inhibition of lncRNA GAS5 alleviates myocardial ischemia–reperfusion injury by reducing the occurrence of apoptosis. It has been recognized that apoptosis is an important feature that influences neuronal tissue damage after SCI (Kawabata et al., 2010); therefore, we hypothesized that lncRNA GAS5 may have an impact on secondary injury by regulating apoptosis. In our study, we found GAS5 to be significantly unregulated in the SCI mice and neurocytes following hypoxia treatment. Moreover, we found that LV-shRNA injection improved functional recovery and reduced the lesion size in these mice. In addition, we found that GAS5 silencing suppressed apoptosis in the SCI mice and neurocytes following hypoxia treatment. These findings indicated that GAS5 might play a significant role in the progression of a secondary injury after SCI. However, the spinal cord is a complex tissue with many cell types, and our data reflects expression changes and functions of lncRNA GAS5 in the whole tissue, rather than in specific cells. This may mistakenly attribute some functions to certain cells. More in-depth research into related types of cells is needed in the future.

As is known, inflammation is a major risk factor for secondary injury after SCI (Bethea et al., 1998). Large amounts of pro-inflammatory cytokines induced by SCI can promote neuronal apoptosis and further aggravate a secondary injury (Lee et al., 2017); therefore, any improvement in inflammatory responses may be an effective strategy for treating SCI. Previous studies have reported that GAS5 acts as a new regulator of the inflammatory response in various inflammatory diseases. For example, Li et al. (2018) have shown that GAS5 ameliorates inflammatory injury in chondrocytes by inhibiting the NF-κB and Notch signaling pathways. Shen et al. (2019) have shown that GAS5 silencing alleviates inflammation in mice with atherosclerosis by upregulating miR-135a. In the present study, we found that GAS5 silencing suppressed the inflammatory response, which indicated that GAS5 improved the secondary injury after SCI by regulating inflammation.

Recent studies have demonstrated that lncRNAs may act as ceRNAs to regulate miRNA expression, thereby modulating the expression of miRNA target genes (Yoon et al., 2012; Kallen et al., 2013). Among these, the reciprocal interaction between GAS5 and miRNAs was widely studied as an important mechanism. For example, Li and Liu (2020) have found that GAS5 suppresses the inflammatory responses of alveolar epithelial cell MLE-12 by targeting the miR-429/DUSP1 axis. Ji et al. (2020) have shown that GAS5 silencing may suppress apoptosis and the inflammatory response of osteoarthritic chondrocytes by targeting the miR-34a/Bcl-2 axis. In this study, we considered whether GAS5 functioned as a ceRNA. Using bioinformatics software, we found that GAS5 has binding sites with several miRNAs that are associated with SCI. QRT-PCR data showed that GAS5 had the most significant effect on miR-93 expression, while there was no distinct change observed in other miRNAs. Subsequently, RIP and RNA pulldown assays further confirmed that GAS5 could directly bind to miR-93.

Previous studies have demonstrated the neuroprotective role of miR-93 in SCI. For example, Yan et al. (2017) have found that miR-93 inhibits the development of neuropathic pain in mice with chronic constriction sciatic nerve injury possibly by inhibiting STAT3-mediated neuroinflammation. Chen et al. (2016) have shown that miR-93 may promote neurite outgrowth in spinal cord neurons from reduced levels of p-ephexin and active RhoA. In fact, one previous study has confirmed that miR-93 is negatively regulated by GAS5 in a hypoxia-induced cardiomyocyte injury model (Du et al., 2019); however, whether silencing GAS5 protects SCI by sponging miR-93 remains unclear. In the present study, we demonstrated that the protective actions of silencing GAS5 on SCI mice and hypoxia-induced HT22 cell injury were reversed by suppressing miR-93. In addition, PTEN was identified as a target gene of miR-93. PTEN, a well-known tumor suppressor, has been identified as a key regulator in neuronal apoptosis. Some previous studies have demonstrated that the inhibition of PTEN could suppress neuronal apoptosis (Cui et al., 2016; Ma et al., 2016). Intriguingly, we identified that GAS5 had a positive correlation with PTEN expression in SCI mice and hypoxia-injured HT22 cells through sponging miR-93. Together with the above data, we drew a preliminary conclusion that GAS5 worked as a ceRNA for miR-93 and subsequently prevented PTEN from degradation by miR-93, thereby inhibiting neuron cell apoptosis and inflammation.

There were still some limitations in this study. To preserve afflicted tissues or rescue injured cells as much as possible in the crushed spinal cord by preventing or alleviating secondary injury is one principle in the treatment of spinal cord injury. However, therapeutics aimed at reducing the secondary injury may also affect some other aspects of the long-term resolution of SCI (Beattie et al., 2002). For example, reduction of excitotoxic cell death early after injury will likely spare more axons, resulting in a decrease of the later glial cell death (Mazzone et al., 2013). Moreover, blocking secondary injury may also influence the reparative responses, such as growth factor production, or even sprouting (Tsai et al., 2008). Although our data suggest that silencing of lncRNA GAS5 plays an important role in improving spinal cord injury, long–term consequences of these findings including effects of distant organs (in particular brain regions) and the natural recovery that appears to occur over time should be taken into account. Moreover, the SCI injury cell model was established in HT22 cells, not in primary cultured neurons. We will evaluate the effects of GAS5 in primary cultured neurons in the future. Some lncRNAs have been found to be therapeutic targets for neural stem cell transplantation and hydrogen sulfide treatment aimed at alleviating SCI (Zheng et al., 2017; Liu et al., 2018). Therefore, lncRNAs could be promising biomarkers for the diagnosis, treatment, and prognosis of SCI.

## Conclusion

The present study has for the first time revealed that silencing GAS5 improved functional recovery and suppressed neuron cell apoptosis and inflammation by regulating the miR-93/PTEN axis in the secondary injury after SCI. Our findings suggest that the GAS5/miR-93/PTEN axis might provide new insights into the prevention and treatment of SCI.

## Data Availability Statement

The original contributions presented in the study are included in the article/Supplementary Material, further inquiries can be directed to the corresponding author/s.

## Ethics Statement

The animal study was reviewed and approved by the Ethics Committee for Experimental Animals of Zhongshan Hospital, Fudan University.

## Author Contributions

HL and ZC conceived and participated in the design of the study. YC and CJ conducted all experiments. YC, CJ, HL, and ZC participated in data analyses. YC and CJ wrote the manuscript. All authors contributed to the article and approved the submitted version.

## Conflict of Interest

The authors declare that the research was conducted in the absence of any commercial or financial relationships that could be construed as a potential conflict of interest.

## Footnotes

^1^https://www.flowjo.com/solutions/flowjo/downloads/previous-versions

^2^https://cm.jefferson.edu/rna22/Precomputed/

^3^http://www.targetscan.org/vert_72/
