# Supplementary figures and images for: Silencing of Long Noncoding RNA Growth Arrest–Specific 5 Alleviates Neuronal Cell Apoptosis and Inflammatory Responses Through Sponging microRNA-93 to Repress PTEN Expression in Spinal Cord Injury
Source: Front Cell Neurosci. 2021 May 14;15:646788. doi: 10.3389/fncel.2021.646788 (PMC8163226; doi:10.3389/fncel.2021.646788)

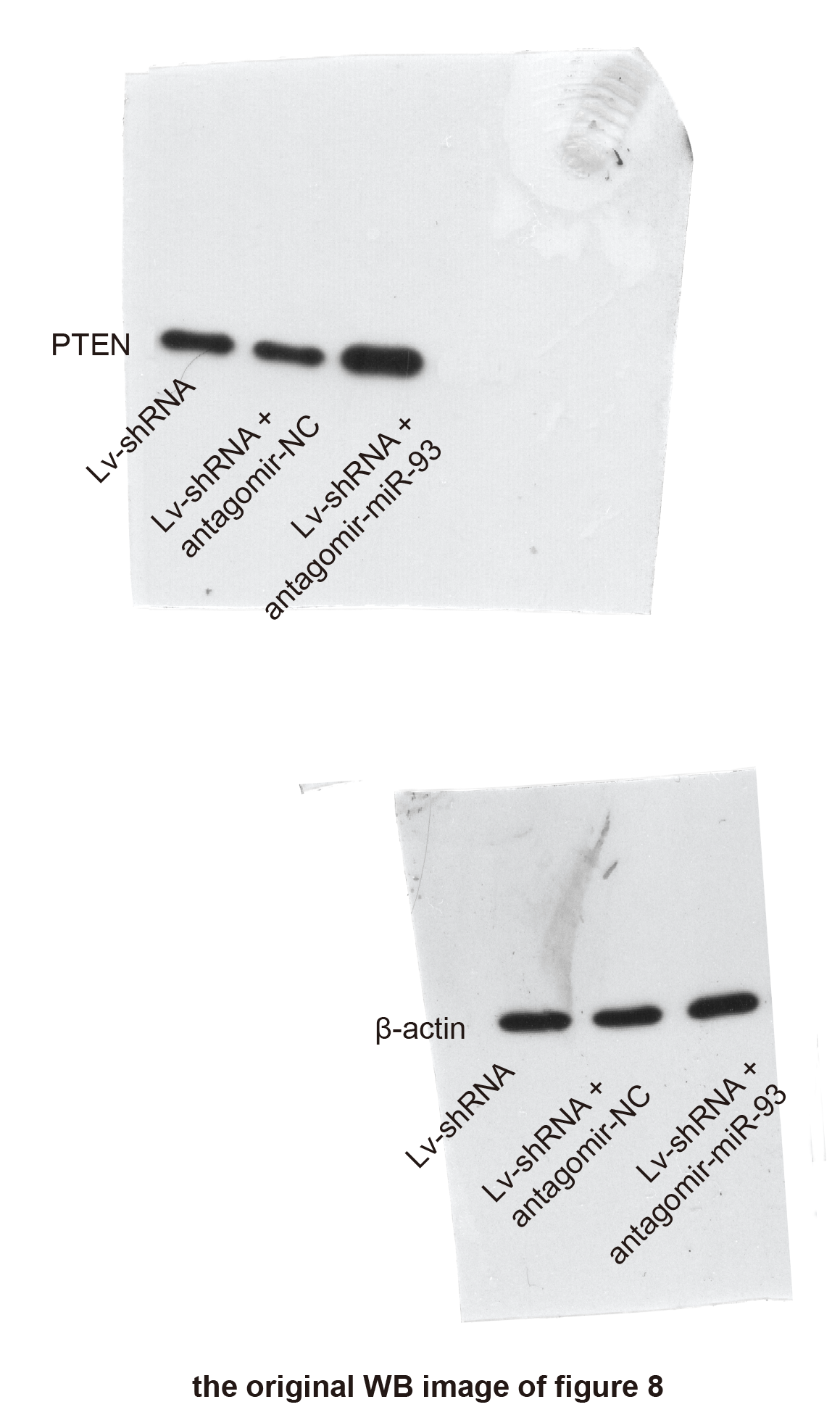

Supplement: Supplementary file 1 [file Data_Sheet_1.ZIP › the original WB image of figure 8.tif]

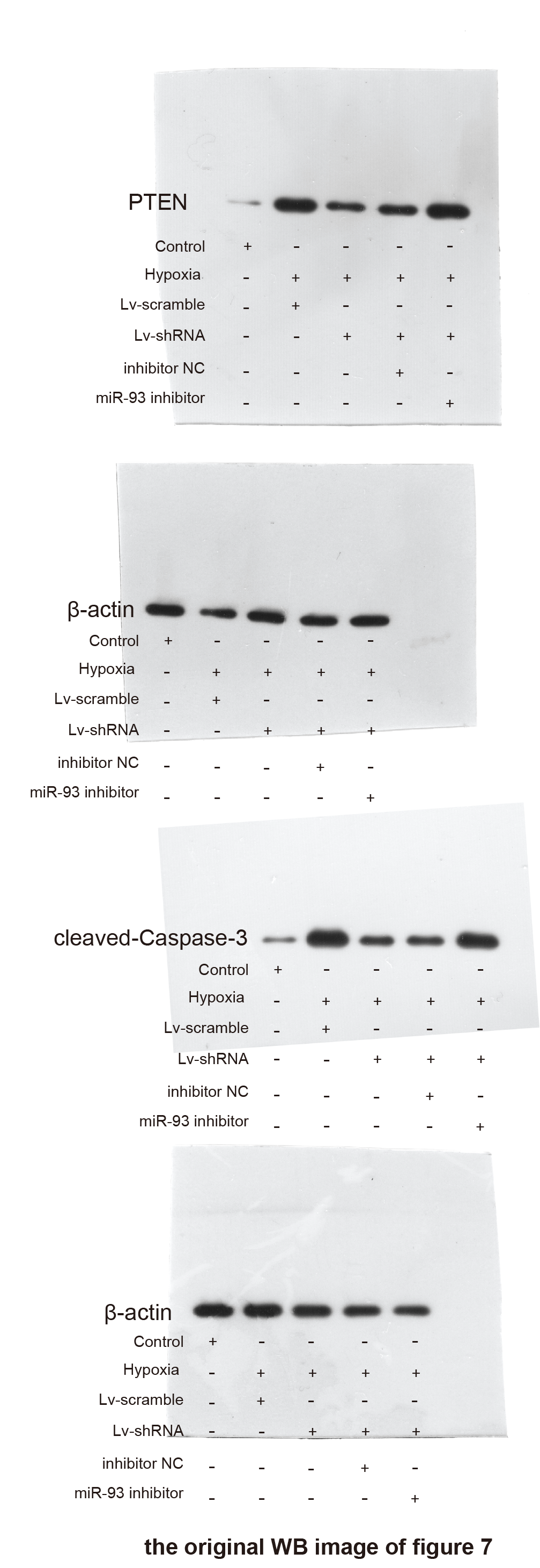

Supplement: Supplementary file 1 [file Data_Sheet_1.ZIP › the original WB image of figure 7.tif]

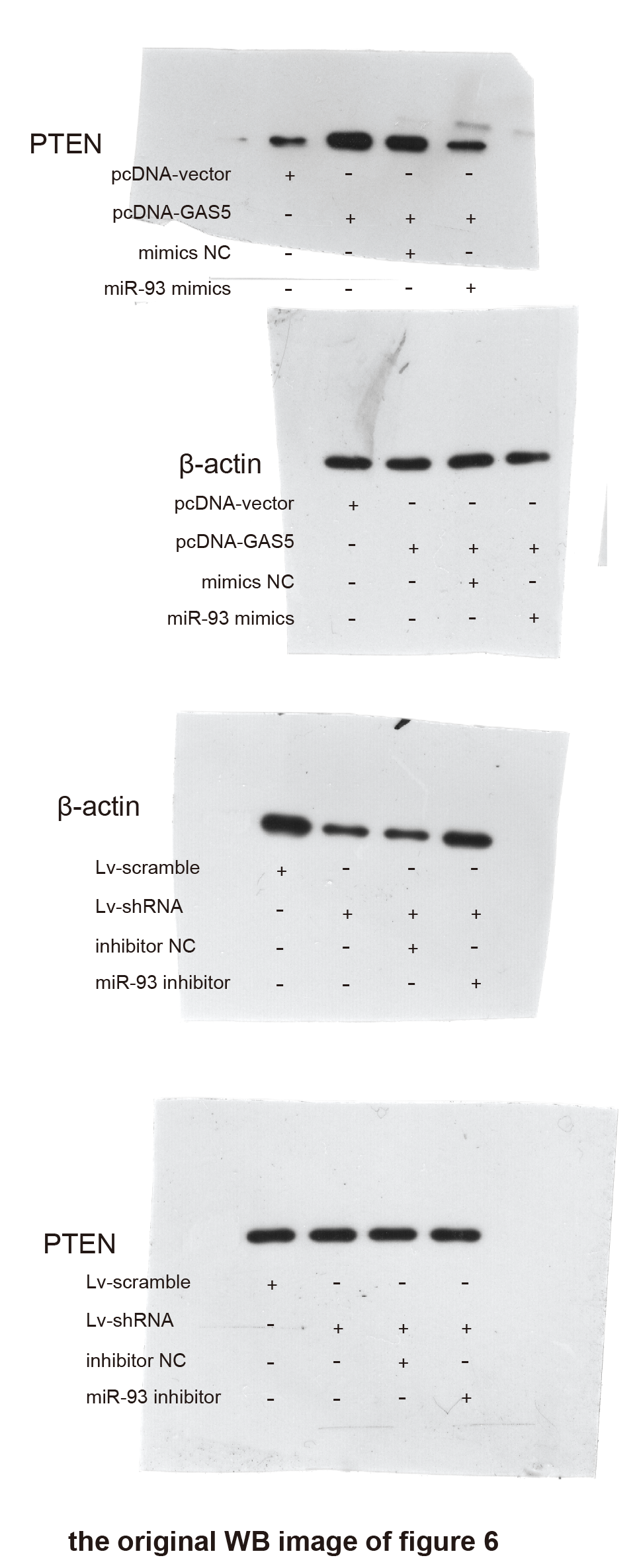

Supplement: Supplementary file 1 [file Data_Sheet_1.ZIP › the original WB image of figure 6.tif]

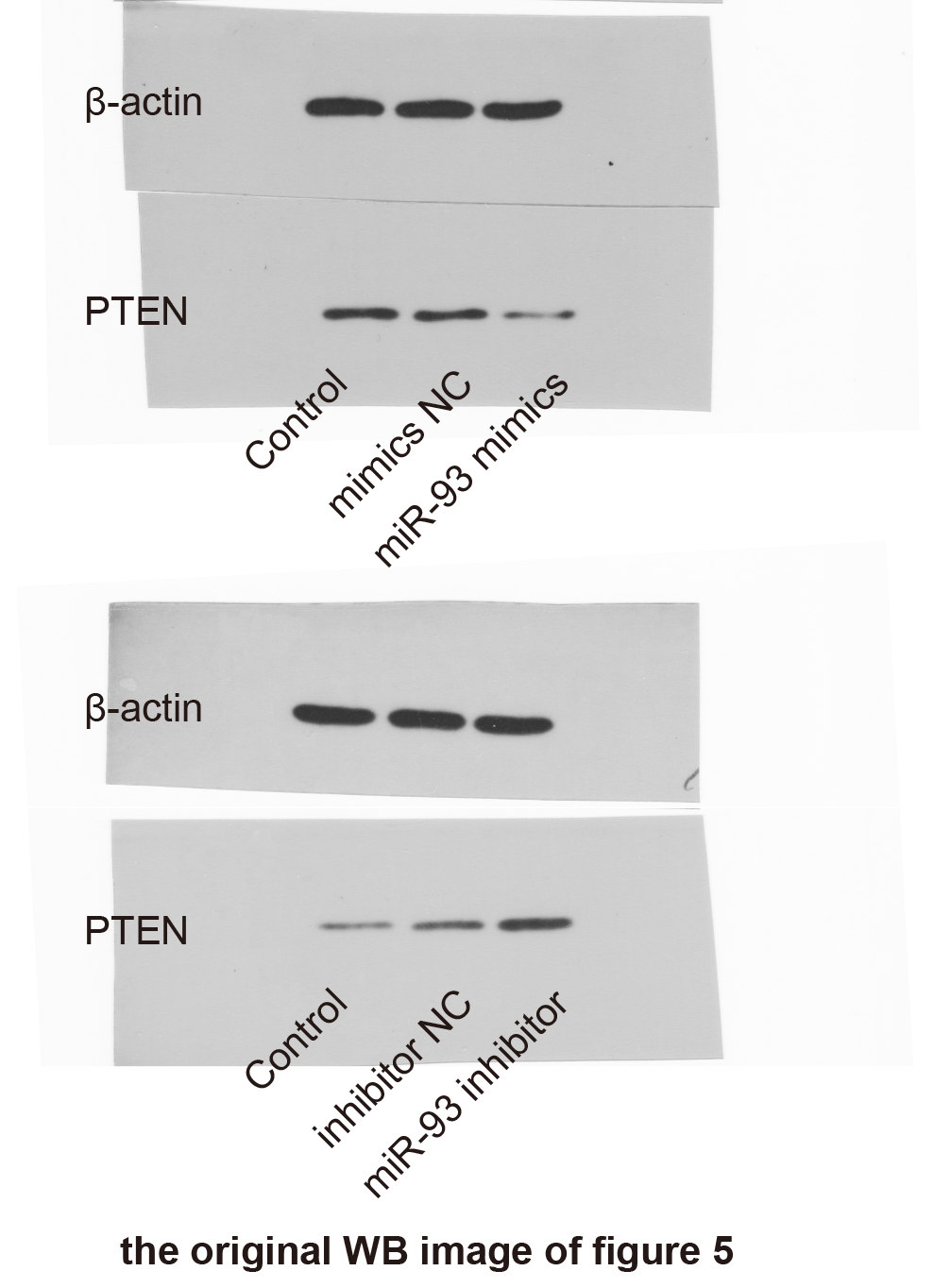

Supplement: Supplementary file 1 [file Data_Sheet_1.ZIP › the original WB image of figure 5.tif]

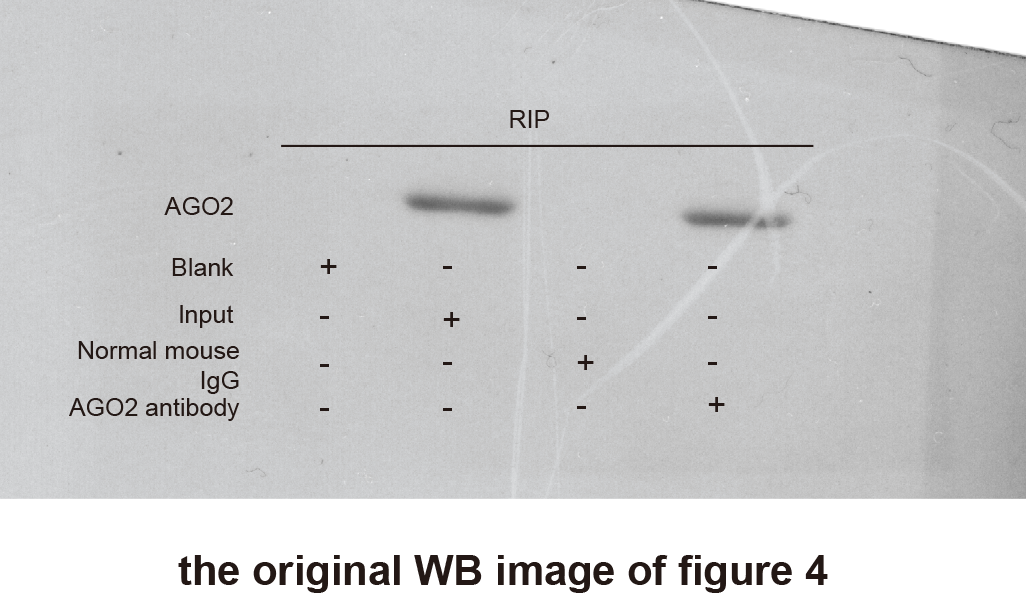

Supplement: Supplementary file 1 [file Data_Sheet_1.ZIP › the original WB image of figure 4.tif]

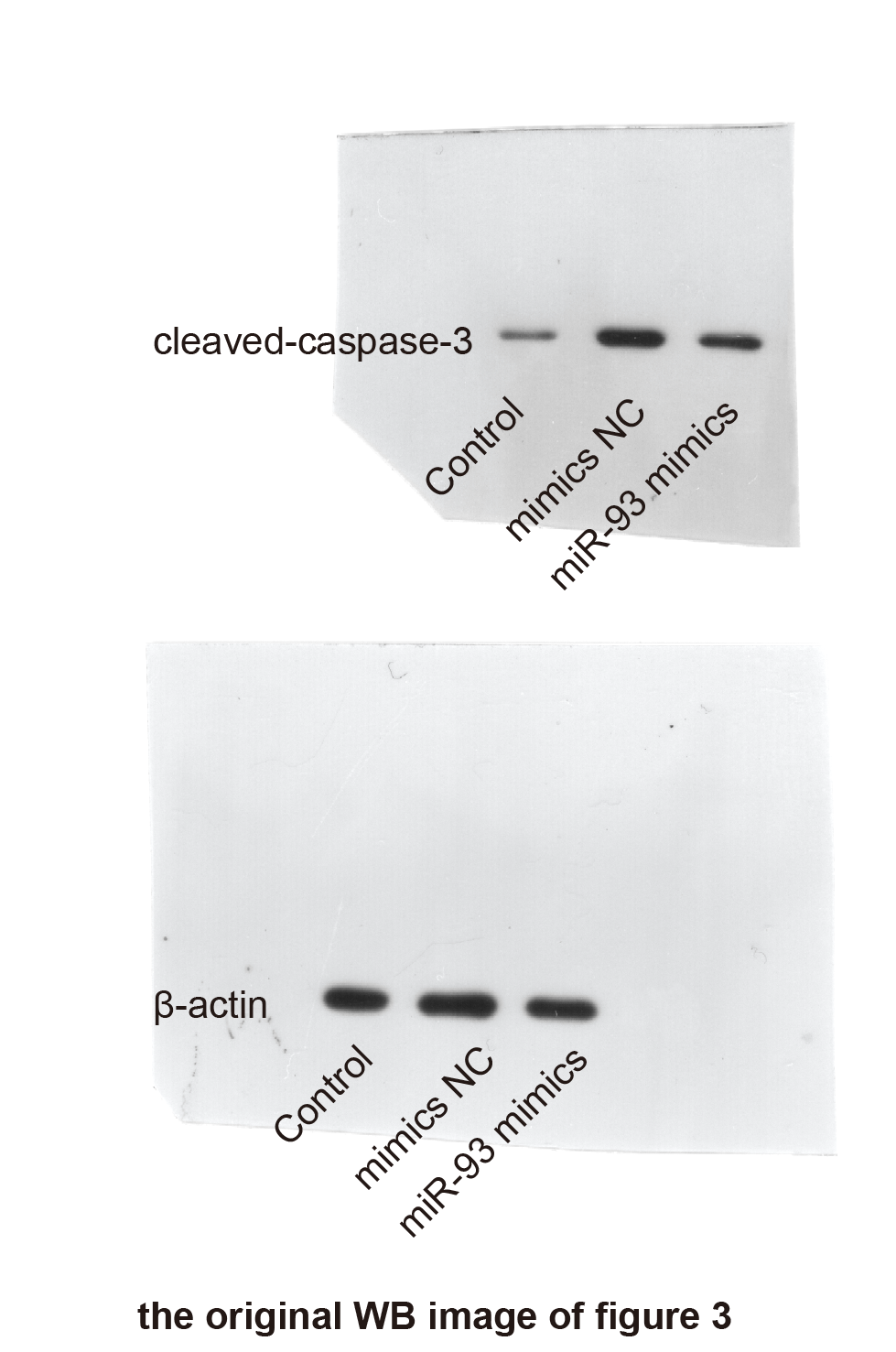

Supplement: Supplementary file 1 [file Data_Sheet_1.ZIP › the original WB image of figure 3.tif]

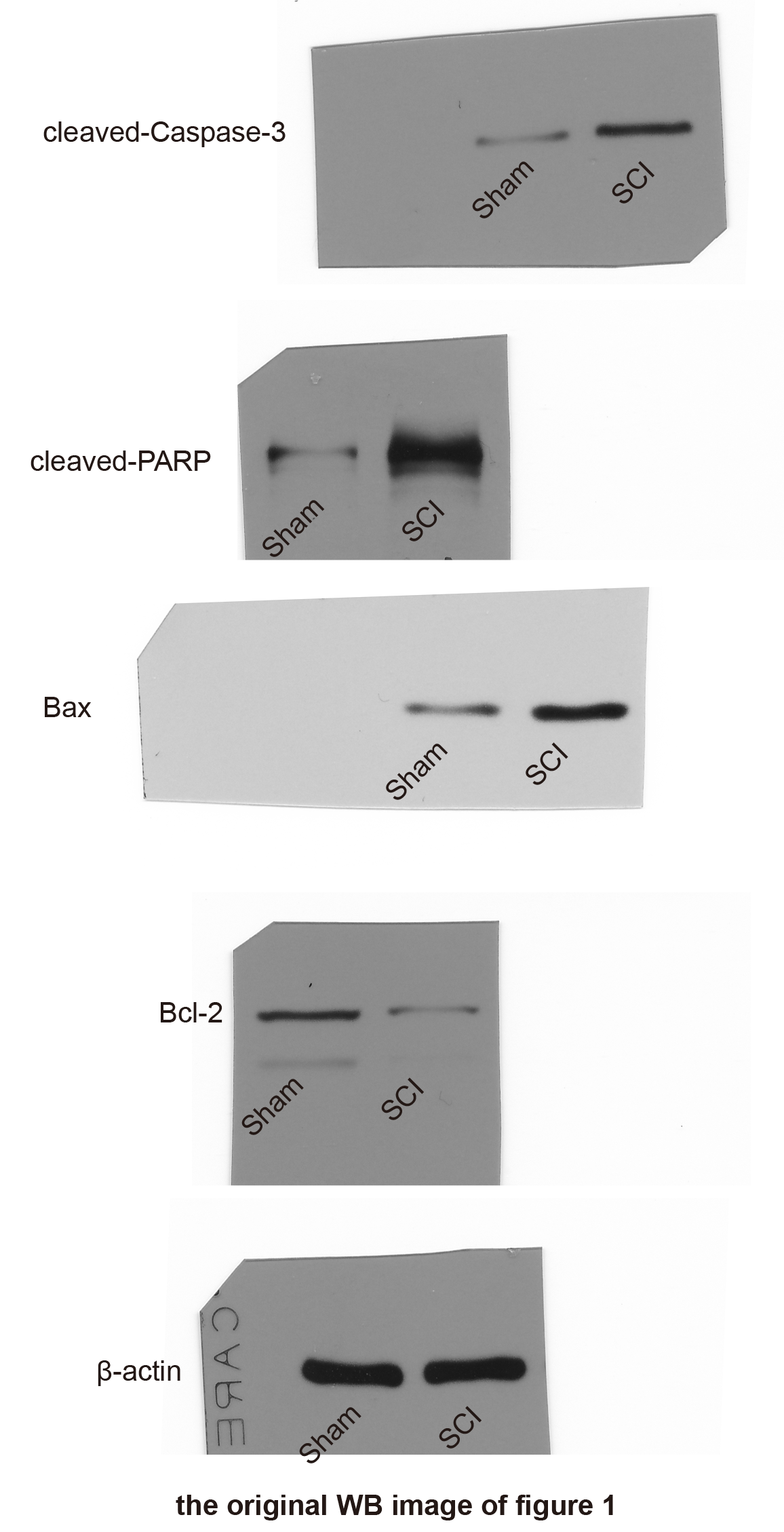

Supplement: Supplementary file 1 [file Data_Sheet_1.ZIP › the original WB image of figure 1.tif]
